# Consistent or inconsistent? The effects of inducing cognitive dissonance vs. cognitive consonance on the intention to engage in pro-environmental behaviors

**DOI:** 10.3389/fpsyg.2022.902703

**Published:** 2022-08-24

**Authors:** Lucia Bosone, Marie Chevrier, Franck Zenasni

**Affiliations:** ^1^Uni Gustave Eiffel, Université de Paris, LaPEA, Versailles, France; ^2^Université Paris Cité and Uni Gustave Eiffel, LaPEA, Boulogne-Billancourt, France

**Keywords:** cognitive dissonance, cognitive consonance, psychological barriers, intention to change, pro-environmental behavior

## Abstract

How do individuals rationalize the cognitive dissonance between their environmental awareness and the maintenance of environmentally unfriendly behaviors? The main goal is to explore the rationalization strategies used by individuals in order to maintain their current behaviors. The secondary goal is to understand if it is possible to induce cognitive consonance, and how this influences intention to change. We present a study (*N* = 222) with three experimental conditions: inconsistency, control, and consistency. The method to induce inconsistency and consistency was inspired by the paradigm of induced hypocrisy. Results demonstrated that induced inconsistency elicits two main barriers in participants: considering the change as unnecessary, and perceiving to lack knowledge about how to change. Induced consistency elicits tokenism, resulting in a licensing effect. However, behavioral intentions did not differ among experimental groups. Results are discussed considering methodological limitations and possible intervening variable.

## Introduction

Despite the high level of public awareness about the environmental impact of human activity, there is a significant gap between the attitudes and the behaviors of individuals toward the protection of the environment. A recent worldwide survey on the public understanding of climate change (Leiserowitz et al., 2021) shows that if the majority of the participants recognize that climate change is happening (91% in France, 90% in the United Kingdom, 80% in the United States), only a small percentage would be ready to participate in a citizen’s campaign urging action to reduce climate change (21% in France, 17% in the United Kingdom, 20% in the United States). If the literature about pro-environmental behavioral change usually focuses on the psychological dimensions motivating individuals to engage in such a change (e.g., Kothe et al., 2019; Yuriev et al., 2020), it is also important, when developing education and communication campaigns, to understand the psychological barriers to individuals’ decision to engage in behavioral change. Numerous studies have analyzed the psychological barriers that prevent individuals from engaging in pro-environmental behaviors (e.g., Lacroix et al., 2019), such as the perception of behavioral change as unnecessary, including the belief in technological solutions, feelings of powerlessness, and denial of responsibility for climate change. People may also fail to act when they feel a lack of knowledge about how to take action to protect the environment, or because they find it difficult to change their habits, or that the costs and constraints of the new behavior are perceived as too high (i.e., conflicting goals and aspirations). To these barriers is added the weight of the norms of people’s surroundings, or the importance of other people’s views on changing one’s behavior (i.e., interpersonal relations). Finally, some individuals consider their efforts sufficient, thus hindering them in adopting new pro-environmental behaviors; this is a phenomenon called “tokenism,” where individuals consider they have done enough and will not do anything more. If such barriers have been identified as determining inaction with regards to different environmental issues (e.g., climate change mitigation; Lacroix et al., 2019; biodiversity conservation; Bosone et al., under revision), it is still not clear which ones are used by individuals to justify their maintenance of the gap between their awareness and their behaviors, which creates cognitive dissonance.

Cognitive dissonance is a situation of incongruence between attitudes and behaviors that lead to a negative affective state characterized by discomfort, tension and physiological arousal (Festinger, 1957). Such negative affective state makes it unpleasant to hold inconsistent attitudes and/or perceptions or to behave inconsistently (e.g., Zanna and Cooper, 1974; Elliot and Devine, 1994). The desire to resolve cognitive dissonance drives individuals either to change their behavior in order to behave consistently, or to change their beliefs so that they are consistent with the current behavior, or yet to justify (i.e., rationalize) such discrepancy to make it bearable (McGrath, 2017).

Individuals who are aware of environmental problems but do not act to prevent them are confronted with this state of discomfort. To justify such a gap, they can engage in rationalization strategies, allowing them to keep their current behaviors while knowing that they are not contributing to the protection of the environment. We argue that some of the aforementioned psychological barriers to environmental action could actually be rationalization strategies to justify the maintenance of a behavioral inconsistency (i.e., cognitive dissonance). Clearly identifying such rationalization strategies could have important applied implications, potentially guiding education and communication programs targeting individuals who are already sensitive to their environmental impact, but who have yet to improve their pro-environmental efforts.

This is the main objective of the present paper: to analyze which barriers are the most highlighted by individuals who are in a state of cognitive dissonance, which can be obtained experimentally by inducing inconsistency (also defined as “induced hypocrisy”; Priolo et al., 2019).

### Induced inconsistency

Induced inconsistency appears to be a paradigm of cognitive dissonance (Aronson et al., 1991; Fointiat, 2004). This technique triggers cognitive dissonance reaction among participants or citizens, to study and induce a behavior change. Two steps are necessary for individuals to become aware of their state of inconsistency (“hypocrisy”) between their beliefs and their actions.

In the first stage of the induced hypocrisy technique, participants of the studies are led to declare or to recognize a normative behavior or a general norm (e.g., presenting a scientific expert delivering a speech stating that researchers are now convinced that human behavior is the source of global warming; Priolo et al., 2016). Recalling the transgression is the second step in the method of induced hypocrisy. Researchers create a situation of dissonance between the previously stated norm and the participant’s behavior, by asking individuals to recall and describe past occasions when they failed to adopt a pro-environmental behavior (Harmon-Jones et al., 2003).

Such induced state of cognitive dissonance can encourage a change of intention as well as observed behavior (such as frequency and amount of donation; Priolo et al., 2016; Odou et al., 2019). However, no study has yet used this technique to observe which psychological barriers become rationalization strategies for individuals in a condition of induced inconsistency. This is thus the main aim of our research. Such a methodology brings us to a second research question: if it is possible to induce inconsistency and thus increase behaviors by asking people to remember how often they failed to adopt pro-environmental behaviors, does that mean that it is also possible to induce consistency? And would this decrease behavior?

### Induced consistency

This second research question poses a methodological dilemma, because if asking individuals to remember how many times they have failed in the past to adopt pro-environmental behavior is an effective technique to induce inconsistency, then asking individuals how many times they have adopted the behaviors in the past could induce consistency. Being this a way used by many researchers to measure past behaviors, before exposing participants to interventions promoting behavioral change, in a pre/post-intervention analysis that is often advocated to assess actual behavioral change (e.g., Abrahamse and Matthies, 2018; Miller, 2021), it would be important to understand whether this could bias the answers of the participants.

Based on past literature, two opposite hypotheses could be argued with regards to a positive vs. negative impact of such induced consistency on behavioral intentions.

On one hand, asking individuals to recall how many times they behaved pro-environmentally could influence their perception of themselves as people who are respectful of the environment, thus affecting their pro-environmental self-identity (Sparks and Shepherd, 1992; Stryker and Burke, 2000; Cook et al., 2002; Christensen et al., 2004). An individual’s self-identity determines the consistency between his or her attitudes and actions, and thus assures the continuity of specific behaviors across different experiences and contexts. Past research has demonstrated how pro-environmental self-identity is a strong determinant of pro-environmental behavior across different areas, such as food consumption (Grewal et al., 2000; Cook et al., 2002), recycling (Mannetti et al., 2004) and in general pro-environmental action (Terry et al., 1999; Fekadu and Kraft, 2001). It is thus possible to suppose that induced consistency could boost pro-environmental self-identity and thus increase individuals’ willingness to engage in even more pro-environmental behaviors. This would also be in line with the principles of the commitment theory (Kiesler, 1971; Girandola, 2005) which explains that once individuals act, they tend to become committed to their action, which creates a consolidation of the underlying attitudes. This would lead us to expect that the more individuals realize they have behaved pro-environmentally, the more they become committed to this, the more they intend to pursue such pro-environmental conduct.

However, the opposite could also be argued: asking participants to recall how much they have already engaged in pro-environmental behaviors could increase tokenism, which is one of the barriers to action (Gifford and Chen, 2017). Indeed, it has been demonstrated that after behaving pro-environmentally, one may feel they “have done enough,” and have acquired a moral license to make less pro-environmental efforts (Kennedy et al., 2009; Nolan and Schultz, 2015; Geng et al., 2016). It is thus possible to suppose that individuals in a condition of induced consistency could feel as if they have already changed their behaviors in favor of the environment enough, and that anything more would be too much to handle. An increase in tokenism could thus decrease their intention to engage in more pro-environmental efforts.

A secondary objective is thus to understand (1) if it is possible to induce cognitive consonance, or consistency, by reminding individuals of their pro-environmental actions, and (2) in which direction induced consistency could influence individuals’ intention to engage in specific individual and collective environmental actions.

## Method

### Participants and procedure

To estimate the sample size needed for this study, we used the effect size found in a recent meta-analysis of the effect of induced hypocrisy (Priolo et al., 2019). The meta-analysis (*k* = 19, *N* = 1,127) shows variation between a low and moderate correlation coefficient for the effect of induced hypocrisy on behavioral intention [*r* = 0.35, 95%CI (0.22, 0.46)]. Because we are also comparing dissonance and consonance in this study, we chose to be more conservative and determine the sample size on a small effect size (*r* = 0.22, corresponding to *f* = 0.226); G*Power indicates a sample size of at least 192 participants to achieve 80% power for an ANOVA analysis with 3 groups and 1 predictor. A total of 225 participants were recruited online, posting the link to the survey on several social media groups, not directly related to environmental issues.

Three were excluded for failing one or two instructional attention checks, leaving a sample of 221 participants (48.4% men and 51.1% women), aged 18–62 years (*M* = 29.4, *SD* = 9.12). Participants voluntarily answered to the survey and gave their agreement for the use of their data; data collection and analyses followed the latest General Data Protection Regulation. Participants were randomly assigned to one of three conditions: induced inconsistency, induced consistency, and a control condition. After giving their agreement, participants were asked to read a text about the consequences of CO_2_ emissions on global warming and biodiversity loss. This is the first step for inducing cognitive inconsistency vs. consistency. The text emphasized the responsibility of each individual in the emission of CO_2_ and therefore the importance of adopting pro-environmental behaviors (e.g., reducing meat consumption, private car use). Then, participants in the inconsistency condition were asked to recall how often they had failed to adopt a list of six pro-environmental behaviors in the previous month [1 = I never fail, 5 = I always fail; α(*N* = 6) = 0.88], while participants in consistency condition were asked to recall how often they adopted these behaviors [1 = Never, 5 = Always when possible; α(*N* = 6) = 0.89]. Participants in the control condition only read the message without being asked about their past behaviors.

All participants were then asked a series of close-ended scale questions about their current emotional state, the barriers they felt that prevented them from improving specific pro-environmental changes to their lifestyle, and their intention to do so. Finally, participants were thanked and fully debriefed about the objective of the study.

### Measures

#### Emotional state

A short version of the dissonance thermometer was first administered in order to check for the effectiveness of the inconsistency and consistency induction (inspired by Priolo et al., 2016): four items measured (on a scale from 1—Not at all to 5—Completely) the participants’ level of negative emotions (i.e., uncomfortable, embarrassed, bothered, and anxious; α = 0.83) and four items measured the participants’ level of positive emotions (i.e., content, good, proud, relaxed; α = 0.86).

#### Psychological barriers

Psychological barriers to action were measured by a scale of 23 items adapted from the DIPB scale (Lacroix et al., 2019), asking participants to think about individual lifestyle changes to reduce carbon footprint, in favor of the environment. Participants were then asked to rate their agreement with the items on a 7 points Likert scale going from 1—Not at all to 5-Completely. Six factors emerged from a Principal Component Analysis, accounting for 74.3% of the variance in the dataset. Two items contributed to more than one factor and were then excluded from further analysis. The rest of the items were computed in order to create the mean score for each factor: the perception of the changes as unnecessary [α(*N* = 3) = 0.76], lacking knowledge [α(*N* = 3) = 0.92], the perception of such changes as being in conflict with their own goals and aspirations [α(*N* = 4) = 0.88], interpersonal relationships [α(*N* = 4) = 0.87], tokenism [α(*N* = 4) = 0.92] and the external attribution of the responsibility for such changes [α(*N* = 3) = 0.66]. The loading values of each factor are presented in Supplementary Table 1.

#### Pro-environmental self-identity

This was measured through the scale of pro-environmental self-identity adapted from Whitmarsh and O’Neill (2010), including four items on a 7-point agreement scale, such as “I think of myself as someone who is very concerned with environmental issues” [α(*N* = 4) = 0.70].

#### Behavioral intentions

Participants were asked to declare to what extent they intend, in the future, to adopt four different pro-environmental behaviors [from 1 Not at all to 7 Completely; α(*N* = 4) = 0.90]: to increase use of eco-friendly modes of transportation, to increase the accuracy of recycling, to buy local products more often, and to buy products with less packaging more often.

## Results

We carried out a MANOVA to test the influence of consistency/inconsistency on positive and negative emotions, the six psychological barriers, pro-environmental self-identity and behavioral intention; all values are reported in Table 1, including power analysis and confidence intervals. When a significant result was obtained, we also carried out Tukey *post-hoc* tests to identify which groups significantly differed.

**TABLE 1:** MANOVA values.

Measure	*F*	*p*	η*_*p*_*^2^	95% CI
Behavioral intentions	2.70	0.069	0.024	[5.07; 5.72]
Positive emotions	9.14***	0.000	0.077	[2.34; 2.77]
Negative emotions	10.34***	0.000	0.086	[2.46; 2.86]
**Psychological barriers**				
Unnecessary change	4.44*	0.013	0.039	[2.35; 2.88]
Conflicting goals	1.93	0.147	0.017	[2.89; 3.45]
Interpersonal relationships	1.39	0.251	0.013	[1.84; 2.27]
Lack of knowledge	9.08***	0.000	0.077	[3.67; 4.36]
Tokenism	5.60***	0.004	0.049	[2.01; 2.63]
Externalization of responsibility	1.99	0.139	0.018	[4.19; 4.75]
Pro-environmental self-identity	2.05	0.131	0.018	[5.15; 5.57]

*p < 0.05, ***p < 0.005.

The decision to present the results of the MANOVA was taken in spite of the fact that all dependent dimensions did not distribute normally (all Shapiro-Wilk’s test yielded a *p* < 0.05, except for the barrier “external attribution,” for which Shapiro-Wilk’s *p* = 0.08). This decision is due to the fact that appropriate non-parametric tests were also carried out (both Generalized Linear Models, and Kruskal-Wallis tests), and all of the tests yielded the same significant and non-significant results. Since results did not vary across different types of tests, we decided for clarity’s sake to report here only the results of the MANOVA.

Before each analysis, we checked for outliers on the dependent variables (by examining the studentized residuals with a Bonferroni test). The only outlier was found on one of the barriers (change not necessary); we thus carried out the analysis to check for any possible difference due to this outlier. Since the analyses did not differ, we present in the following section all the analyses including the one outlier. The raw data supporting the conclusions of this article will be made available by the corresponding author, without undue reservation.

### Behavioral intentions

The induction of consistency and inconsistency did not significantly influence behavioral intentions. However, there is a tendency to significance [*F*_(2, 219)_ = 2.71, *p* = 0.07, η*_*p*_*^2^ = 0.02]: individuals in the consistency condition reported lower intentions (*M* = 4.91, *SD* = 1.76) than individuals in the inconsistency condition (*M* = 5.39, *SD* = 1.43) and in the control condition (*M* = 5.39, *SD* = 1.19). Tukey *post-hoc* tests did not show any significant difference; this tendency to significance suggests that further studies, with different methodologies, are needed (as presented in the discussion).

### Positive and negative emotions

The induction of consistency and inconsistency had a significant effect on positive [*F*_(2, 219)_ = 9.14, *p* < 0.001, η*_*p*_*^2^ = 0.08] as well as negative emotions [*F*_(2, 219)_ = 10.34, *p* < 0.001, η*_*p*_*^2^ = 0.08].

More precisely, individuals in the inconsistency condition reported lower positive emotions (*M* = 2.55, *SD* = 1.02) and higher negative emotions (*M* = 2.66, *SD* = 0.98) than individuals in the control condition (PE: *M* = 3.11, *SD* = 0.84; Tukey *p* = 0.007; NE: *M* = 1.93, *SD* = 0.83, Tukey *p* < 0.001) and in the consistency condition (PE: *M* = 3.15; *SD* = 1.03; Tukey *p* < 0.001; NE: *M* = 2.22, *SD* = 0.92, Tukey *p* = 0.005). The reported negative and positive emotions felt by individuals in the consistency condition and in the control condition did not significantly differ.

### Psychological barriers

**FIGURE 1:**
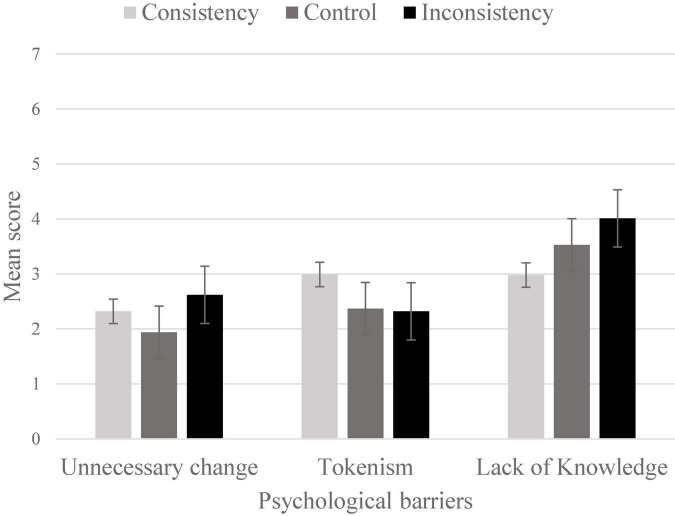
The effects of induced consistency/inconsistency on three psychological barriers. Error bars are standard errors.

The induction of consistency and inconsistency had a significant effect on individuals’ ratings of three of the six barriers to action identified: perception of the change as unnecessary, lacking of knowledge, and tokenism. Only these three barriers are discussed further in this section, and presented in Figure 1; however, the means and standard deviations for each barrier are reported in Table 2.

**TABLE 2:** Means, standard deviations of dependent variables for all conditions (only measures with significant MANOVA test are reported).

Measure	Consistency	Control	Inconsistency
			
	*M* (*SD*)	*M* (*SD*)	*M* (*SD*)
Behavioral intention	4.91 (1.76)	5.39 (1.19)	5.39 (1.43)
Positive emotions	3.15_a_ (1.03)	3.11_a_ (0.84)	2.55_b_ (1.02)
Negative emotions	2.22_a_ (0.92)	1.93_a_ (0.83)	2.66_b_ (0.98)
**Psychological barriers**			
Unnecessary change	2.32 (1.26)	1.94a (1.07)	2.62b (1.29)
Conflicting goals	2.79 (1.34)	2.92 (1.21)	3.17 (1.31)
Interpersonal relations	1.83 (0.96)	2.07 (1.07)	2.06 (1.04)
Lack of knowledge	2.98_a_ (1.53)	3.53 (1.51)	4.01_b_ (1.75)
Tokenism	2.99_a_ (1.68)	2.37_b_ (0.96)	2.32_b_ (1.37)
External attribution	4.11 (1.43)	4.43 (1.05)	4.47 (1.25)
Pro-environmental self-identity	5.66 (0.99)	5.48 (1.05)	5.36 (0.94)

The means of the same dependent variable having different subscripts in the different experimental groups (subscript a vs. subscript b) differ significantly at p < 0.05 as indicated by Tukey post hoc tests. For instance, the means for positive emotions in the consistency and control group, with the subscript a, differ significantly from the mean for positive emotions in the inconsistency condition, with the subscript b.

#### Perception of the change as unnecessary

Results shown that the ratings of the change as unnecessary were higher for individuals in the Inconsistency condition (*M* = 2.62; *SD* = 1.29) than the ratings of the individuals in the control condition (*M* = 1.94, *SD* = 1.07, Tukey *p* = 0.01). No significant difference was found between individuals in the inconsistency and in the consistency conditions (*M* = 2.32, *SD* = 1.26).

#### Lacking knowledge

The ratings of lacking knowledge as a barrier to action were higher for individuals in the Inconsistency condition (*M* = 4.01; *SD* = 1.76) than the ratings of the individuals in the consistency condition (*M* = 2.98; *SD* = 1.53; Tukey *p* < 0.001), but no significant difference was found between individuals in these conditions and the individuals in the control condition (*M* = 3.53, *SD* = 1.51).

#### Tokenism

Tokenism as a barrier to action was rated higher for individuals in the consistency condition (*M* = 2.99; *SD* = 1.68) than the ratings of the individuals in the control condition (*M* = 2.37; *SD* = 0.96; Tukey *p* = 0.05) and individuals in the inconsistency condition (*M* = 2.32, *SD* = 1.37, Tukey *p* = 0.006). The ratings of individuals in the inconsistency condition did not differ from the ratings of individuals in the control condition.

### Pro-environmental self-identity

No significant difference was found when comparing the ratings of pro-environmental self-identity by individuals in the inconsistency vs. control vs. consistency conditions.

## Discussion

The first objective of the present work was to identify which among the psychological barriers are also rationalization strategies that individuals use to justify, and thus accept, the dissonance between their environmental awareness and their inaction. To do so, we induced in a third of our sample a condition of cognitive dissonance using the paradigm of induced hypocrisy, by focusing their attention on the importance of the adoption of pro-environmental behaviors at the individual level and then asking them to recall how often they failed to adopt pro-environmental behaviors. Such induction was effective as demonstrated by the influence of our manipulation on positive and negative emotions felt by individuals in the inconsistency condition and individuals in the control condition.

As with regards to the psychological barriers, the comparison between the answers of individuals in the Inconsistency condition and individuals in the Control condition reveals that the consideration of the change as unnecessary is the barrier that is significantly more invested by the individuals when they are in a situation of cognitive dissonance.

The second objective of the present work was to verify whether it is also possible to induce a state of “cognitive consonance,” corresponding to a state of consistency between the beliefs and the behaviors of the individuals, and whether this would modify their intention to engage in further pro-environmental efforts. To this purpose, the comparison between the answers of the individuals in the Consistency condition and the individuals in the Control condition with regards to their positive and negative emotions does not suggest that inducing Consistency has an effect on the emotional states of the individuals. However, the same comparison shows how inducing Consistency increases individuals’ perception of having engaged in enough pro-environmental effort so that they don’t need to do more (“tokenism”). This is in line with past work on the “licensing effect” (e.g., Burger et al., 2022), which defines a phenomenon where after behaving pro-environmentally, individuals may feel they have acquired a moral license to make less pro-environmental efforts (e.g., Geng et al., 2016). Considering past literature on spillover effects (Elf et al., 2019) as well as the consolidation effect of commitment (Kiesler, 1971; Girandola, 2005), further research should investigate more deeply what are the possible factors determining whether being aware of one’s own pro-environmental behaviors result in licensing or spillover effects, such as individuals’ concern with the environment. Indeed, individuals’ pre-existing beliefs about climate and the environment, and their awareness of the problematic influence of human activity on them (e.g., Eisenack et al., 2014), could have modulated the effect of our induction, resulting in a stronger or weaker consistency and inconsistency. For instance, individuals’ environmental self-identity could be a valuable moderator of the influence of consistency/inconsistency, especially considering that our data demonstrated that self-identity was not influenced by the induction. It would be possible to suppose for instance that the induction of inconsistency/consistency has an effect only for individuals with low environmental self-identity, as high environmental self-identity is strongly connected with pro-environmental behavior (Wang et al., 2021).

Present data showed that inconsistency activates a specific rationalization strategy that is considering the change as unnecessary, whereas consistency activates tokenism. This difference might suggest that while considering the change as unnecessary is indeed a rationalization strategy to justify the maintenance of a *status quo* which is inconsistent with personal beliefs, tokenism is rather a thought emerging from reflecting on one’s own behaviors which could bias research on this topic. Further, lacking knowledge also differs between induced consistency and inconsistency, however, the fact that neither differ significantly from the control condition prevent us from concluding whether it is a barrier activated by inconsistency, or whether this corresponds to a real lack of knowledge. Further research could include a measure of knowledge (e.g., Prévot et al., 2018), and problem awareness as we suggested above, before the induction of consistency/inconsistency.

These effects of induced inconsistency and consistency do not seem to consequently influence individuals’ behavioral intentions, as the comparison between the control condition and the conditions where inconsistency or consistency were induced does not yield significant differences. Indeed, only a tendency to significance emerges when comparing individuals’ intentions in the three conditions. The non-significant difference between the Inconsistency and the Control condition seems in contrast with past literature about induced hypocrisy, which demonstrated that triggering a state of cognitive dissonance increased behavioral intentions (for a review, see Priolo et al., 2019). This could be linked to three possible reasons: the first reason has to do with the method used to induce cognitive dissonance. Indeed, in the present study, we did not ask participants to express their belief about the importance of individuals’ pro-environmental actions, but rather we explicitly focused their attention on it by asking them to read a text about this. This might have limited the cognitive dissonance felt by individuals, as our method of induction did not trigger a contrast between their beliefs and their actions, but rather between what they should do and what they failed to do. A pre-test would be needed to further confirm that the texts really increased the salience of their normative believes. Moreover, we induced consistency and inconsistency by asking individuals about six specific behaviors, while past research has demonstrated that reporting too many transgressions can reduce the hypocrisy effect (Fointiat et al., 2008; Stone and Fernandez, 2011). Further research could try to replicate present findings with a more traditional method to induce hypocrisy.

A second reason could depend on the fact that the behaviors targeted (mobility choices, recycling, purchasing) usually have the features of behavioral habits. Indeed, although many behaviors originate from thinking and considering possible alternatives, individuals do not go through such deliberate decisional process for actions that are repeated regularly and frequently (Aarts et al., 1998). When the same behavior is adopted many times and very often, such as buying specific products or using a specific transportation mode (e.g., driving a car), it can become a habit. Habitual behaviors are extremely resistant to permanent change (e.g., eating habits), and others are only changed slowly, over decades, making them resistant even to priming and attitude change (Verplanken and Aarts, 1999). It would thus be expected that individuals with strong recycling, purchasing and driving habits might not be as sensible to cognitive dissonance as other individuals with weaker habits. Future research could focus on measuring how the motivating effect of triggering cognitive dissonance to promote pro-environmental attitudes and behavior could vary depending on the strength of specific behavioral habits.

A third possibility might be the fact that behavioral intention are measured after psychological barriers, and it is possible that thinking about the reasons why individuals do not behave pro-environmentally might offer them a justification for it, and thus decrease the influence of induced inconsistency. Further research should thus explore whether the effects of induced inconsistency and consistency on behavioral intentions are stronger if psychological barriers are measured after the intentions.

It is important to point out that this study offers preliminary findings on the risk of inducing consistency by asking individuals how often they engaged in pro-environmental behaviors in the period before participating to the study, which is a technique used frequently to establish a baseline of pro-environmental behaviors. Indeed, data shows that individuals in the Consistency condition reported higher rates of tokenism than individuals in the inconsistency or control conditions. However, in the current study we only used subjective measures of consistency/inconsistency and intention. Further research could investigate further this effect combining objective measures, such as physiological measures of negative emotions induced by cognitive dissonance (e.g., Colosio et al., 2017), as well as implicit measures, such as implicit association tests (e.g., Panzone et al., 2016).

Overall, present findings offer important theoretical contributions and potential practical implications. On one hand, identifying the rationalization strategies used by individuals to maintain environmentally harmful behaviors in spite of evidence on the necessity to change is a very important knowledge in order to guide the development of education and communication programs. Indeed, an effective intervention motivates citizens by pushing on the right levers, but also by tackling the possible cognitive barriers to change.

Present findings concern individuals in an induced state of cognitive dissonance, and thus need further empirical confirmation—concerning individuals in a “natural” state of cognitive dissonance—to corroborate their applied impact. However, it is possible to propose some insights on how these data could inform education and communication programs. For instance, our data demonstrated that individuals in a condition of cognitive dissonance consider the change proposed as unnecessary more strongly than individuals in the other conditions. This finding points to the fact that the perception of the change as unnecessary might indeed be the main barrier to be tackled by education and communication programs. In order to prevent such a rationalization strategy, messages used in such programs should focus more on the positive consequences of changing one’s own behaviors, aiming at improving individuals’ perception of the effectiveness and utility of the changes promoted. Communication strategies could also be used to nudge individuals toward considering the change as effective and necessary. For instance, it could be possible to match the message framing to the behaviors promoted. Indeed, it has been demonstrated (Bosone et al., 2015) that gain-framing improves perceived effectiveness of additive behaviors (i.e., doing something new, such as enroll in a mobility challenge), whereas loss-framing improves perceived effectiveness of subtractive behaviors (i.e., reducing a harmful behavior, such as reducing the use of one’s own car to commute). Another example could be to nudge individuals by using a narrative format, rather than a numerical one, to present data about the behavioral change proposed, as current research has demonstrated that narratives are more effective than statistics in increasing perceived response-efficacy (Bosone et al., under revision). These communication nudging techniques could improve individuals’ consideration of the utility of changing their behaviors, thus preventing rationalization strategies to set in motion.

On the other hand, present findings warn about the risk of inducing cognitive consonance by asking individuals to recall their engagement in pro-environmental behaviors before a behavioral change intervention, as this could bias its effectiveness. This is the first time that such a concept of cognitive consonance has been tested experimentally and will need further analysis to propose possible solutions to measure behavioral baselines before behavioral change interventions.

## Data availability statement

The raw data supporting the conclusions of this article will be made available by the authors upon request, without undue reservation.

## Author contributions

LB conceived the study, participated in its design and coordination, as well as to data collection and analysis, and wrote the first draft of the article. MC participated in the design of the study, the data collection and analyses, as well as in the writing, and formatting of the article. FZ participated in the data analyses, as well as in the revision and formatting of the manuscript. All authors read and approved the final manuscript.
