# Supplementary material for: Consistent or inconsistent? The effects of inducing cognitive dissonance vs. cognitive consonance on the intention to engage in pro-environmental behaviors
Source: Front Psychol. 2022 Aug 24;13:902703. doi: 10.3389/fpsyg.2022.902703 (PMC9454017; doi:10.3389/fpsyg.2022.902703)
Supplement: Supplementary file 1 [file Data_Sheet_1.DOCX]

Supplementary Material

Consistent or inconsistent? The effects of inducing cognitive dissonance versus cognitive consonance on the intention to engage in pro-environmental behaviors

# Supplementary Data - Methods

## Recall of the normative behaviors in environmental issues:

All greenhouse gases are naturally present in the atmosphere, and the greenhouse effect contributes to life on Earth. When it is too much, the greenhouse effect causes global warming.

Human activity causes enormous concentrations of carbon dioxide (CO2) in the atmosphere, being responsible for two thirds of the additional greenhouse effect and causing environmental catastrophes such as climate change and biodiversity loss.

CO2 is massively produced by the combustion of fossil fuels (transport, heating, energy production...). Deforestation, (destruction of forests to replace them with fields) also releases CO2 into the atmosphere. Humans are also increasing methane production through intensive animal husbandry, but above all through the most widely grown cereal on Earth: rice. Rice fields release a very large amount of methane.

These examples remind us that each of us is responsible, at our own level, for the increase of CO2 in the atmosphere and global warming, this is called our "Ecological footprint". This footprint can be reduced by modifying our behaviors, such as reducing the consumption of meat and the use of private cars, improving recycling...

## Induction of cognitive dissonance:

*Question: How often have you FAILED to perform the following behaviors in the past month?*

*Answers: 1 - I never fail 2 - I rarely fail 3 - I sometimes fail 4 - I often fail 5 - I always fail*

1. I separated waste and recyclable material
2. I limited my consumption of disposable products (plastic bags, plastic packaging, etc.)
3. I limited my energy consumption (by unplugging and/or turning off devices/lights/heaters when not in use, etc.)
4. I opted for an environmental mode of transportation instead of using a car
5. I bought regionally produced products
6. I re-used plastic and glass containers

## Induction of cognitive consonance:

*Question: How often have you performed the following behaviors in the past month?*

*Answers: 1 -Never 2 -Rarely 3 - Sometimes 4 -Often 5 - Always when possible*

1. I separated waste and recyclable material
2. I limited my consumption of disposable products (plastic bags, plastic packaging, etc.)
3. I limited my energy consumption (by unplugging and/or turning off devices/lights/heaters when not in use, etc.)
4. I opted for an environmental mode of transportation instead of using a car
5. I bought regionally produced products
6. I re-used plastic and glass containers

## Emotional state:

*Question: How do you feel?*

*Answers: 1 - Does not correspond at all to 5 - Correspond completely*

1. Content
2. Uncomfortable
3. Embarrassed
4. Good
5. Bothered
6. Proud
7. Anxious
8. Relaxed

## Psychological barriers:

*Answers: 1 - Strongly disagree to 7 - Strongly agree*

1. *^^[[1]](#footnote-1)^^There's not much point in me making this change because I feel confident that technological innovators will solve environmental problems.
2. Humans are powerless when it comes to saving the earth, so there is no need to change.
3. These problems are so far in the future that there is no need to act.
4. There's a need for change because I believe that a serious environmental problem exists.
5. What happens at the industrial level makes my changing insignificant.
6. Making this change would interfere too much with my other goals in life.
7. I'm concerned that this change will take up too much of my time.
8. I can't change because I'm invested in my current lifestyle.
9. These issues are important to me but it's too hard to change my habits.
10. *I haven't changed because I'm afraid this wouldn't work.
11. Making this change would be criticized by those around me.
12. I would be letting certain people down if I made this change.
13. I'm worried that my friends would disapprove if I made this change.
14. If I made the necessary change, I would probably be embarrassed when others noticed what I was doing.
15. There's so much information out there that I am confused about how to make this change.
16. I don't understand enough of the details about how to make this change.
17. I'd like to change but I'm not sure where to begin.
18. The pro-environmental efforts that I currently engage in make further changes unnecessary.
19. I've already made sacrifices to solve environmental problems, so there is no need for me to do more.
20. I have previously made important effort in this, so there is no need for me to make further changes.
21. My environmental actions already make enough of a difference.
22. It's not fair for me to change when really it's industry that's causing the majority of environmental problems.
23. The government should make it easier for me to change, if it really has the best interest of the environment in mind.

## Behavioral intentions:

*Answers: 1 - Not at all to 7 - Completely*

1. I intend to increase my use of eco-friendly modes of transportation (public transportation, cycling).
2. I intend to increase the accuracy of my recycling.
3. I intend to buy local products more often.
4. I intend to buy products with less packaging more often.

## Pro-environmental self-identity:

*Answers: 1 - Not at all to 7 - Completely*

1. I think of myself as an environmentally-friendly consumer.
2. I think of myself as someone who is very concerned with environmental issues.
3. I would be embarrassed to be seen as having an environmentally-friendly lifestyle.
4. I would not want my family or friends to think of me as someone who is concerned about environmental issues.

# Supplementary Tables

## Table 1. Factor loadings for the DIPB items on the PCA analysis and fit indices

| Items | PCA loadings | | | | | |
| --- | --- | --- | --- | --- | --- | --- |
|  | 1 | 2 | 3 | 4 | 5 | 6 |
| **Factor 1: Conflicting goals and aspirations** |  |  |  |  |  |  |
| Making this change would interfere too much with my other goals in life. | **.78** | .12 | .19 | .04 | .26 | .12 |
| I’m concerned that this change will take up too much of my time. | **.83** | .10 | .13 | .16 | .17 | .04 |
| I can’t change because I’m invested in my current lifestyle. | **.77** | .14 | .19 | .14 | .24 | .01 |
| These issues are important to me but it’s too hard to change my habits. | **.82** | .02 | .16 | .18 | .06 | .15 |
| *I haven’t changed because I’m afraid this wouldn’t work. | **.48** | .05 | .45 | .21 | .15 | .18 |
| **Factor 2: Tokenism** |  |  |  |  |  |  |
| The pro-environmental efforts that I currently engage in make further changes unnecessary. | .12 | **.83** | .15 | -.11 | .15 | -.03 |
| I’ve already made sacrifices to solve environmental problems, so there is no need for me to do more. | .07 | **.92** | -.03 | -.10 | .02 | .01 |
| I have previously made important effort in this, so there is no need for me to make further changes. | .14 | **.90** | .03 | -.11 | .04 | .03 |
| My environmental actions already make enough of a difference. | -.01 | **.87** | -.01 | -.13 | -.11 | -.04 |
| **Factor 3: Interpersonal relationships** |  |  |  |  |  |  |
| Making this change would be criticized by those around me. | .13 | -.07 | **.81** | .04 | .07 | -.04 |
| I would be letting certain people down if I made this change. | .22 | .05 | **.77** | .08 | .15 | .07 |
| I’m worried that my friends would disapprove if I made this change. | .17 | .10 | **.78** | .22 | .16 | .06 |
| If I made the necessary change, I would probably be embarrassed when others noticed what I was doing. | .13 | .08 | **.81** | .25 | .14 | .09 |
| **Factor 4: Lack of knowledge** |  |  |  |  |  |  |
| There’s so much information out there that I am confused about how to make this change. | .15 | -.20 | .23 | **.87** | .08 | .13 |
| I don’t understand enough of the details about how to make this change. | .16 | -.17 | .20 | **.82** | .18 | .16 |
| I’d like to change but I’m not sure where to begin. | .21 | -.23 | .17 | **.79** | .17 | .12 |
| **Factor 5: Unnecessary change** |  |  |  |  |  |  |
| *There’s not much point in me making this change because I feel confident that technological innovators will solve environmental problems. | .40 | -.05 | .31 | -.05 | **.49** | -.00 |
| Humans are powerless when it comes to saving the earth, so there is no need to change. | .32 | -.07 | .26 | .07 | **.69** | .10 |
| These problems are so far in the future that there is no need to act. | .21 | -.00 | .32 | .13 | **.77** | .12 |
| There’s a need for change because I believe that a serious environmental problem exists (reversed item). | .13 | .13 | -.04 | .23 | **.77** | -.06 |
| **Factor 6: Externalization of responsibility** |  |  |  |  |  |  |
| What happens at the industrial level makes my changing insignificant. | .32 | -.24 | .22 | -.06 | .25 | **.69** |
| It’s not fair for me to change when really it’s industry that’s causing the majority of environmental problems. | .16 | .21 | .07 | .33 | .44 | **.62** |
| The government should make it easier for me to change, if it really has the best interest of the environment in mind. | .02 | .03 | -.02 | .27 | -.20 | **.79** |

The items preceded by * were dropped in further analyses due to poor loadings

1. The items preceded by * were dropped in further analyses due to poor loadings [↑](#footnote-ref-1)
